# A Case Report: Achalasia Presenting With Eating Disorder in an Adolescent

**DOI:** 10.1192/bjo.2026.11903

**Published:** 2026-06-30

**Authors:** Ezgi Deniz Yazici, Aram Yaseri, Harshini Prasanna Kumar, Abdulgafar Yusuf, Hafijur Rahman

**Affiliations:** Birmingham Women’s and Children’s NHS Foundation Trust, Birmingham, United Kingdom

## Abstract

**Aims:**

Achalasia is a rare oesophageal motility disorder in children and adolescents, presenting with vomiting, regurgitation, dysphagia, weight loss, and anxiety around eating. These features can closely resemble restrictive eating disorders or functional vomiting syndromes, leading to diagnostic uncertainty. Delayed diagnosis is well recognised and may result in severe malnutrition, aspiration, and secondary psychiatric morbidity. We describe an adolescent whose prolonged illness was initially managed within eating disorder pathways before achalasia was identified as the underlying cause.

**Methods:**

At 12 years of age, the patient developed eating difficulties in the context of a known anxiety disorder. She reported progressive dysphagia, described as an acidic sensation in the throat and food sticking, alongside regurgitation, reduced appetite, and avoidance of eating at school. Vomiting increased over time, oral intake declined, and significant weight loss ensued.

She was admitted for investigation of possible physical causes. A barium swallow study was attempted but poorly tolerated and completed via nasogastric tube, resulting in inadequate visualisation of the oesophagus. Despite this limitation, the study was reported as normal and no gastroenterology follow up was arranged. Ongoing vomiting and nutritional compromise led to management within eating disorder services and admission to an inpatient eating disorder unit. Atypical anorexia nervosa was the initial working diagnosis.

With structured nutritional rehabilitation, she initially approached weight restoration and was generally engaged in treatment. A previously undisclosed history of self harm emerged, linked to eating related guilt and emotional distress, and fluoxetine was commenced. Although vomiting reduced in frequency, urges persisted and dietary adherence remained inconsistent. She was later diagnosed with ADHD and commenced on methylphenidate.

**Results:**

She subsequently deteriorated, with renewed weight loss, reduced dietary adherence during home leave, and worsening physical symptoms including regurgitation, nocturnal cough, and recurrent chest infections. Symptoms were attributed to reflux and rumination syndrome, and management remained conservative. Progressive nutritional compromise and recurrent infections necessitated nasogastric feeding and detention under the Mental Health Act. Further imaging demonstrated marked oesophageal dilatation with distal narrowing, confirming achalasia. She underwent laparoscopic Heller myotomy, after which regurgitation, vomiting, and chronic cough resolved. Postoperatively, she achieved sustained weight restoration and tolerated oral intake without dysphagia.

**Conclusion:**

This case highlights how achalasia can closely mimic eating disorder presentations when early investigations are inconclusive. In adolescents with persistent dysphagia, regurgitation, vomiting, and weight loss refractory to eating disorder treatment, early reconsideration of gastrointestinal pathology may prevent prolonged psychiatric admissions and significant medical morbidity.

